# ﻿What drives the reduction of hindwings in Galerucinae sensu stricto (Insecta, Coleoptera, Chrysomelidae)? A case study based on the Taiwanese fauna

**DOI:** 10.3897/zookeys.1252.142772

**Published:** 2025-09-19

**Authors:** Chi-Feng Lee

**Affiliations:** 1 Applied Zoology Division, Taiwan Agricultural Research Institute, 189 Chung-Cheng Road, Taichung 413008, Wufeng, Taiwan Taiwan Agricultural Research Institute Taichung Taiwan

**Keywords:** *
Apterogaleruca
*, Citizen science, *
Furusawaia
*, *
Hirtigaleruca
*, leaf beetles, *
Lochmaea
*, *
Paraplotes
*, *
Shairella
*, *
Sikkimia
*, *
Taiwanoshaira
*

## Abstract

Most Taiwanese species of Galerucinae s. str. have been revised taxonomically in recent years. In Taiwan, reduction of hindwings occurs in 37 species in eight genera. A citizen science-led fieldwork project (Taiwan Chrysomelid Research Team, TCRT) targeting the biology of galerucine species has produced many specimens for morphological and taxonomic study and allowed the development of evolutionary scenarios to explain hind-wing reduction in the following genera: *Paraplotes* Laboissière, *Lochmaea* Weise, *Shairella* Chûjô, *Furusawaia* Chûjô, *Sikkimia* Duviver, *Taiwanoshaira* Lee & Beenen, *Apterogaleruca* Chûjô, and *Hirtigaleruca* Chûjô. The results reveal that the drivers of the reduction of hindwings vary across different galerucine genera in Taiwan based on character complexes involved in the transformations.

## ﻿Introduction

Wing size, venation, and form can vary inter- and intraspecifically in many chrysomelids. In addition to the full-winged (macropterous) flying morph, there are several stages of flightless (wing-reduced) morphs. Quantifying stages of wing reductions is challenging. Furth (1980) recognized four stages: macropterous = fully winged; brachypterous = wings reduced (at least one-half of the length of the abdomen), such that flight is not possible; micropterous = rudimentary wing pads or only slightly developed (less than one-half of the abdominal length); and apterous = no vestiges of wings remaining.

Southwood (1962) showed that the level of migratory movement is positively correlated with the instability of habitats and that the prime evolutionary value of migration is in the colonization of changing or temporary habitats. His data on wing polymorphisms in Coleoptera indicated that the flightless morphs are associated with permanent habitats and the flying morphs with temporary, environmentally changing, or unstable habits. He concluded that wing reduction optimizes survival in isolated, stable habitats. Roff (1990) found that decreased environmental heterogeneity favored the evolution of flightlessness based on a number of studies on the variation of wing dimorphism and habitat type and wing morph in the North American Orthoptera. The incidence of flightlessness increases with altitude and latitude but it is not exceptionally high on oceanic islands compared to mainland areas. However, these treatments tended to be monothetic, focusing on single factors to explain wing loss across both taxonomic and ecological boundaries. It is evident that diverse sets of ecological and evolutionary circumstances lead to the loss of flight (Wagner and Liebherr 1992).

Insect juvenile hormone (JH) regulates flight-wing development. Increased levels of JH lead to juvenile characters, i.e., brachyptery, and lower levels to adult characteristics, such as macroptery. In cases of alary polyphenism, abiotic environmental factors may influence the concentration of JH or other morph-determining hormones prior to adult eclosion. The most commonly reported stimulus affecting morph determination is photoperiod. Biotic factors such as high population densities also induce alary polyphenism. In addition, abiotic and biotic factors may interact to determine flight apparatus development (Wagner and Liebherr 1992).

Cox (2004) searched and reviewed literature that provides information on the ability of flight in different subfamilies of Chrysomelidae. Apterism has been observed in species of Eumolpinae, Lamprosomatinae, Chrysomelinae, Galerucinae (Galerucini and Alticini) (Cox 2004), and Cassidinae (Chaboo 2000; Shin and Chaboo 2012). Few species of Taiwanese chrysomelids have been recorded as flightless, including *Odontoedon taiwanus* Ge & Daccordi, 2013 (in Ge et al. 2013) in Chrysomelinae, *Asiorestia taiwana* Kimoto, 1996, *Manobia inhumeralis* Kimoto, 1997 (in Kimoto and Takizawa 1997; replacement name for *M. humeralis* Kimoto, 1996), *Longitarsus bicoloriceps* Chûjô, 1937, *L. hohuanshanus* Kimoto, 1970, *L. ishikawai* Kimoto, 1970, *Ivalia bella* (Chen, 1934), *I. uenoi* (Kimoto, 1970), *Batophila acutangula* Heikertinger, 1921, and *B. taiwanica* Döberl, 2010, and females of *Clavicornaltica mizusawai* Suenaga & Yoshida, 2016 in Alticini; and many species in Galerucinae s. str. (see below).

This study focuses on both brachyelytrous species of Galerucinae s. str. (Beenen and Jolivet 2008) and species with only reduced hindwings. Most Taiwanese species of Galerucinae s. str. have been studied taxonomically since 2009. Sufficient material and data have been collected by citizen scientists conducting host plant surveys and studying adult habitat associations to formulate hypotheses about the loss of wings. Winglessness in galerucines was documented in some species of *Lochmaea* Weise (Lee 2019) and *Shairella* Chûjô (Lee and Beenen 2017; Lee 2022; Lee and Huang 2023), females of *Paraplotes* Laboissière (Lee 2015), all species of *Furusawaia* Chûjô (Lee and Bezděk 2021), *Sikkimia* Duviver (Lee and Bezděk 2016), and *Taiwanoshaira* (Lee and Beenen 2020). Two brachelytrous genera, *Apterogaleruca* Chûjô and *Hirtigaleruca* Chûjô, have not yet been studied in detail, however, basic information is available. A comprehensive evolutionary scenario for the reduction of hindwings in Taiwanese species of Galerucinae s. str. is proposed. Our study emerged from the ongoing data collection by a citizen-science inventory of leaf beetles of Taiwan, led by myself and Hsing-Tzung Cheng (鄭興宗). Our “Taiwan Chrysomelid Research Team” (TCRT) was started in 2005 and has yielded a great deal of specimens (deposited at TARI) and associated data, and many publications (see next section). Therefore, we provide a brief history of this initiative.

## ﻿Material and methods

### ﻿Overview of the Taiwan Chrysomelid Research Team

**Figure 1.**
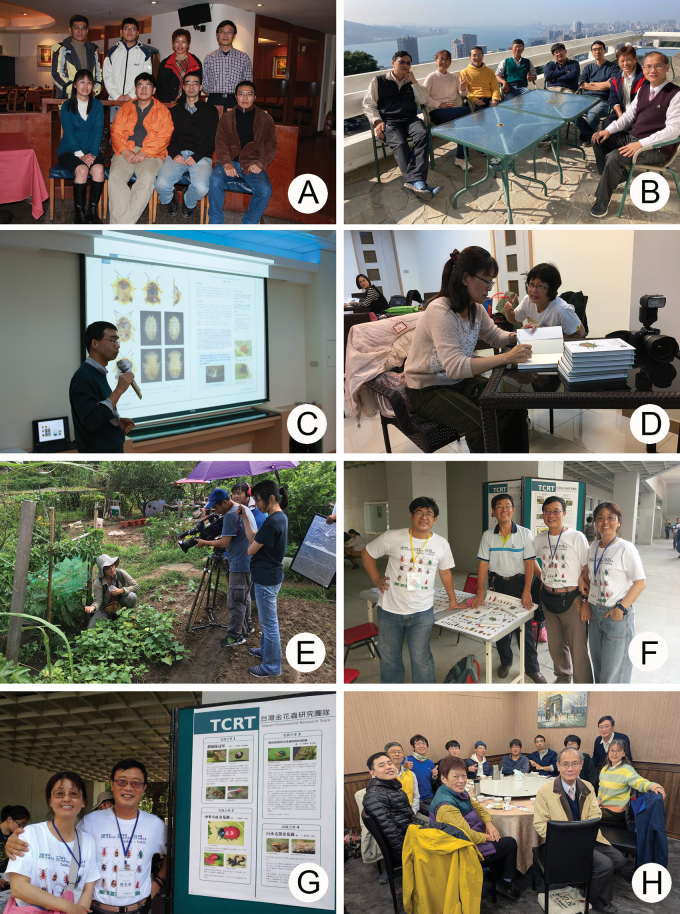
Historical photographs of the TCRT**A** first annual meeting of TCRT in 2008 (from left): Hsuei-Hon Han, Su-Fang Yu, Chih-Kai Yang, Chi-Feng Lee, Hsueh Lee, Hsing-Tzung Cheng, Mei-Hua Tsou, Hou-Jay Chen **B** annual meeting of TCRT in 2010 (from left): Mei-Hua Tsou, Su-Fang Yu, Hou-Jay Chen, Jung-Chang Chen, Chi-Feng Lee, Hsing-Tzung Cheng, Hsueh Lee, Min-Der Chen **C** Hsing-Tzung Cheng introduced the third volume of the book “The Chrysomelidae of Taiwan” during a forum for celebrating the tenth anniversary of TCRT**D** Su-Fang Yu autographed the book at the forum **E** Su-Fang Yu worked as actress on the TV program “Discovery” on the DaAiVideo (大愛電視) **F** exhibit during the 39^th^ Annual Meeting of Taiwan Entomological Society in 2018 (from left): Chi-Feng Lee, Wen-Chuang Liao, Mei-Hua Tsou, Su-Fang Yu **G** Su-Fang Yu and Mei-Hua Tsou stood besides the poser during the meeting in 2018 **H** annual meeting in 2025 (from left): Yen-Cheng Hsu, Jung-Chang Chen, Yi-Ting Chung, Hseuh Lee, Shiang-Lien Yang, Yi-Chia Chiu, Chi-Feng Lee, Hsing-Tzung Cheng, Min-Der Chen, Chen-Han Ma, Mei-Hua Tsou, Su-Fang Yu.

The Taiwan Chrysomelid Research Team was organized by myself and Hsing-Tzung Cheng (鄭興宗) during 2005. Mr Cheng made detailed inventories and photographs of insects and developed a website, “Insect Observation at Sishou Hills” (Cheng 2004). We focused on leaf beetles as the study group and recruited amateurs as a research team. The aim was to inventory all chrysomelid species in Taiwan. In addition to publishing taxonomic works, printing books for educating citizens was also an important goal. The first volume of “The Chrysomelidae of Taiwan” was published in 2007. One hundred easily recognized species were featured (Lee and Cheng 2007). The first paper based on the research team’s collection was published in the same year (Hayashi and Lee 2007). Annual meetings began in 2008 (Fig. 1A), with eight attendees: Su-Fang Yu (余素芳), Chi-Feng Lee (李奇峯), Hsing-Tzung Cheng (鄭興宗), Hou-Jay Chen (陳厚潔), Hsuei-Hon Han (韓學宏), Chih-Kai Yang (楊智凱), Hsueh Lee (李雪), and Mai-Hua Tsou (曹美華). The name “Taiwan Chrysomelid Research Team (TCRT)” was used in Lee and Beenen’s paper (2009) for the first time. In the same year (Lee and Staines 2009), a new species was named for one of TCRT members who first collected the new species. The second book volume was published in 2010 (Lee and Cheng 2010). Two additional members, Jung-Chang Chen (陳榮章) and Min-Der Chen (陳銘德) joined the TCRT in the same year (Fig. 1B). Mr Chen was very active in collecting leaf beetles and made great contributions to the study of *Lochmaea* Weise (Lee 2019). The third book volume was published in 2016 (Lee et al. 2016). Subsequently, we organized a forum to celebrate the publication of the third volume and the tenth anniversary of TCRT (Fig. 1C, D). The story of TCRT was reported on the TV program (Tzu Chi DaAiVideo大愛電視) during the same year (Discovery 2016) (Fig. 1E) and contributed to a popular-science program (PTSKIDS小公視) the same year (Follow me go! 2016). We set up a stand for introducing TCRT and sold posters during the 39^th^ Annual Meeting of the Taiwan Entomological Society in 2018 (Fig. 1F, G). The role of “Citizen Science” in academic studies was addressed by me in 2019 on a TV program (Tzu Chi DaAiVideo大愛電視) (Humanities Lecture 2019). TCRT made the first video to introduce interesting leaf beetles of Taiwan to the public (TCRT in Action! 2019). TCRT has attempted to observe the complete life cycles of leaf beetles the room temperature.

We cooperated with students and faculty at universities for some species living in alpine habitats. For example, *Chrysolina laeviguttata* Chûjô, 1958 is the only member of the genus living in such environments (in Alishan 阿里山). The study on its biology was completed in collaboration with students and faculty at the National University of Tainan with access to thermostatically controlled incubators (Luo et al. 2021).

Recently, five new members joined TCRT, including Yen-Cheng Hsu (徐彥承), Yi-Ting Chung (鍾奕霆), Shiang-Lien Yang (楊庠廉), Yi-Chia Chiu (邱奕家), and Chen-Han Ma (馬承漢) (Fig. 1H). To date, 89 taxonomic papers have been published from the inventory of TCRT.

### ﻿Collection and observation methods

Although this article integrates my previous studies, all Taiwanese species of wingless galerucines were studied using the following procedures. Researchers investigated or collected wingless species at various localities during night hours to determine which species are present. When specimens were located, researchers observed them to determine host plants. When host plants were found, searches were conducted at different localities at night to document patterns of distribution and activity. Observations were made at the same place once or twice each month to investigate patterns of occurrence of adults and larvae. Live females were collected for laboratory rearing to document complete life cycles under controlled conditions.

## ﻿Results

We found that different evolutionary scenarios for the reduction of hindwings occur in different genera. We present each different morphological aspect of wing reduction under the genus accounts below.

### ﻿Genus *Lochmaea* Weise – a case for the reduction of hindwings resulting from adaptation to alpine environments

**Figure 2.**
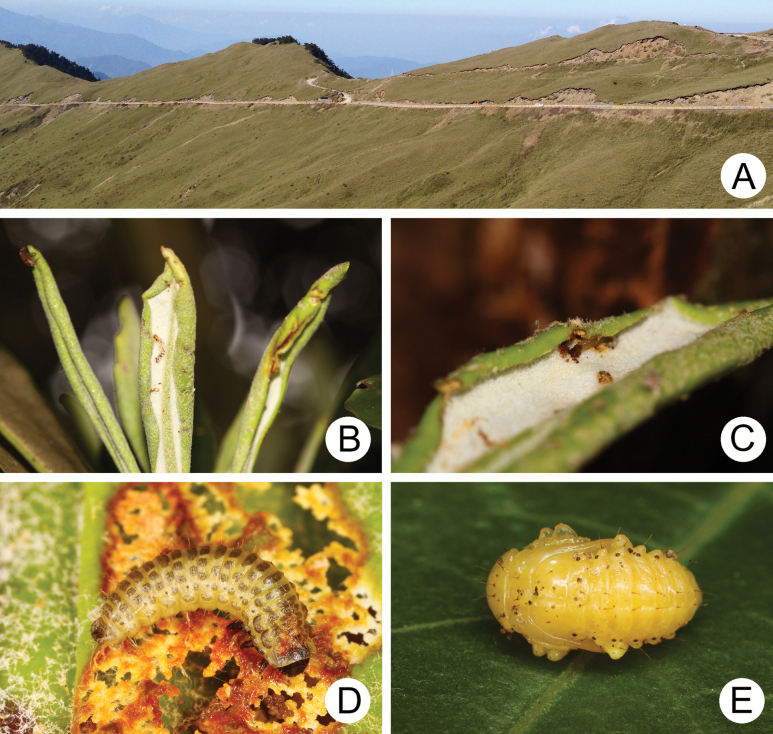
*Lochmaea tsoui* Lee, 2019 **A** Central Cross-Island Highway **B** feeding marks made by mining first-instar larvae **C** first-instar larva concealed under coiled leaf **D** third-instar larva **E** pupa. (after Lee 2019)

Beenen and Jolivet (2008) listed several brachelytrous chrysomelids in alpine regions and suggested that brachelytrous species are relatively more numerous in these habitats. However, the best evidence to support the reduction of hindwings resulting from selection of alpine environments is to compare both winged and wingless species in the same genus, which allows testing across different habitats in monophyletic taxa. In Taiwan, alpine environments are above ~2800−2900 m elevation and are characterized by strong winds and low temperatures, which can be defined by subalpine coniferous vegetation (Zelený 2019). Only short grasses and small trees with thick leaves prevail in these areas (Fig. 2A).

*Lochmaea* is a good candidate for comparative analysis because it comprises in Taiwan two winged and three wingless species (Lee 2019). Members of the genus are univoltine. Larvae and adults of *Lochmaea* feed on leaves of various species of *Rhododendron* (Ericaceae) at different localities. Several young larvae (first instar) were collected from *R. pseudochrysanthum* Hayata in the same area in Kunyang (昆陽, 3050 m, May 10, 2009) (Fig. 2B, C) and transferred to the laboratory for rearing. Mature larvae (Fig. 2D) burrowed into the soil and built underground chambers for pupation (Fig. 2E) on the 15^th^ day (May 25). Adults emerged from the soil after 24 days (June 28). Twenty larvae transformed successfully into adults. Among them, 18 were identified as *L. lesagei* Kimoto, 1996 (winged) and the other two as *L. smetanai* Kimoto, 1996 (wingless).

**Figure 3.**
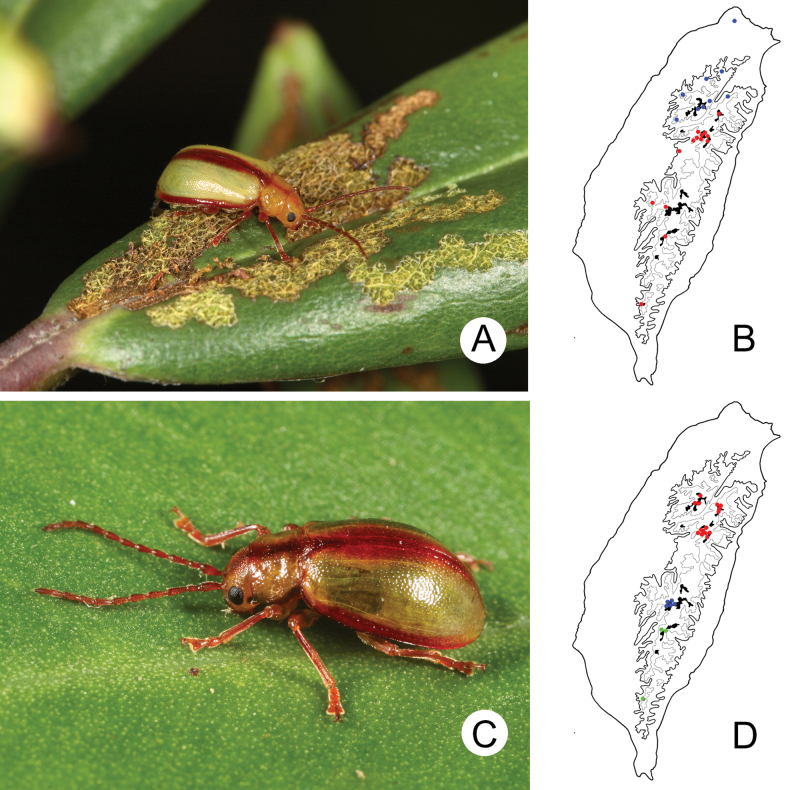
The genus *Lochmaea* Weise **A***L. tsoui*, a winged species **B** distribution of winged species of *Lochmaea* in Taiwan **C***L. chen*, a wingless species **D** distribution of wingless species of *Lochmaea* in Taiwan. Solid line: 1000 m, broken line: 2000 m, black areas: 3000 m; different colors represent different species. (after Lee 2019)

Adults are nocturnal and active on host plants at night. However, they don’t crawl down and shelter on the ground during the daytime. Thus, adults can be collected effectively by beating host plants during the day or night, and they can be trapped using Malaise traps. A number of adults of *L. lesagei* were collected in one Malaise trap set at Yuanfeng (鳶峰, 2756 m) from 2001 to 2006. Records indicated that adults were active from April to November and overwintered from December to March. Lee (2019) studied more than 520 specimens. Adults of *Lochmaea lesagei* (Fig. 3A) and *L. tsoui* Lee, 2019 are winged. They occur not only in alpine areas but also in lower elevation mountains above 1000 m (Fig. 3B). However, the three wingless species (*L. smetanai*, *L. cheni* Lee, 2019, and *L. jungchani* Lee, 2019 (Fig. 3C)) were only collected from alpine areas (Fig. 3D). The distributions of these two species groups indicated that reduction of hindwings similar to that of elytra results from adaptation to alpine environments, with winged forms occurring in intermediate elevations.

### ﻿Genus *Paraplotes* Laboissière – a case of reduction of hindwings in nocturnal species

**Figure 4.**
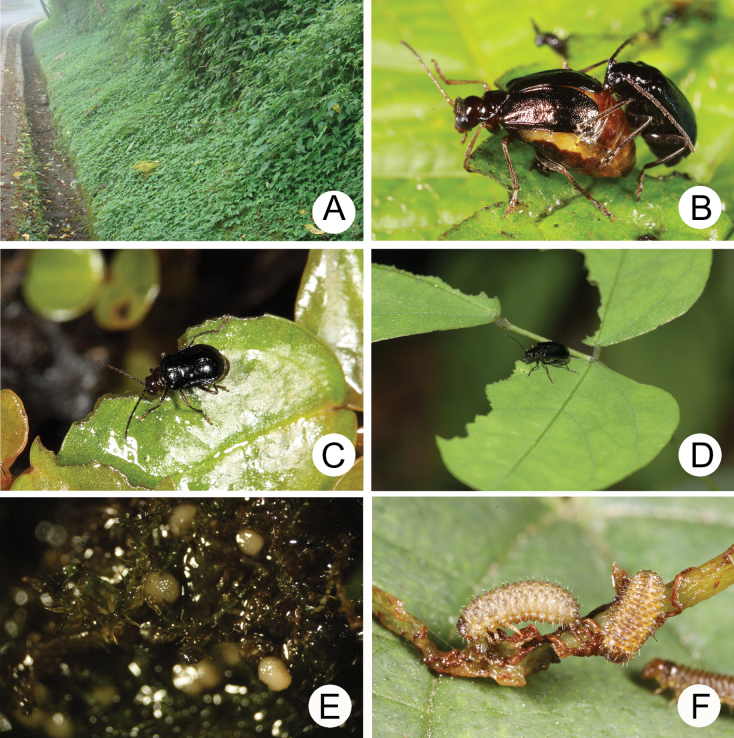
The genus *Paraplotes* Laboissière **A** typical microhabitat **B** pair of adults **C** male of *Paraplotes taiwana* feeding on the leaves of *Pilea rotundinucula* at night **D** male of *Paraplotes yuae* feeding on the leaves of Dumasia villosa subsp. bicolor at day **E** eggs **F** larvae. (Lee 2015)

Among those orders of insects in which both winged and flightless species occur, both sexes are most frequently flightless in six orders, females in nine orders, and in two orders the male is the predominant flightless sex (Roff 1990). Roff (1990) suggested that loss of flight is favored in females because it permits greater allocation of resources to egg production, but that flight is retained in males because it increases the probability of finding a mate. Females of all Taiwanese species of *Paraplotes* are physogastric (Fig. 4B) and brachelytrous, while males have normal abdomens (Fig. 4B–D) and elytra (Fig. 6A). This phenomenon is extremely rare among chrysomelids. Moreover, in *Paraplotes* females appear to be brachyelytrous, whereas they are apterous in *Clavicornaltica* and *Metacycla*.

**Figure 5.**
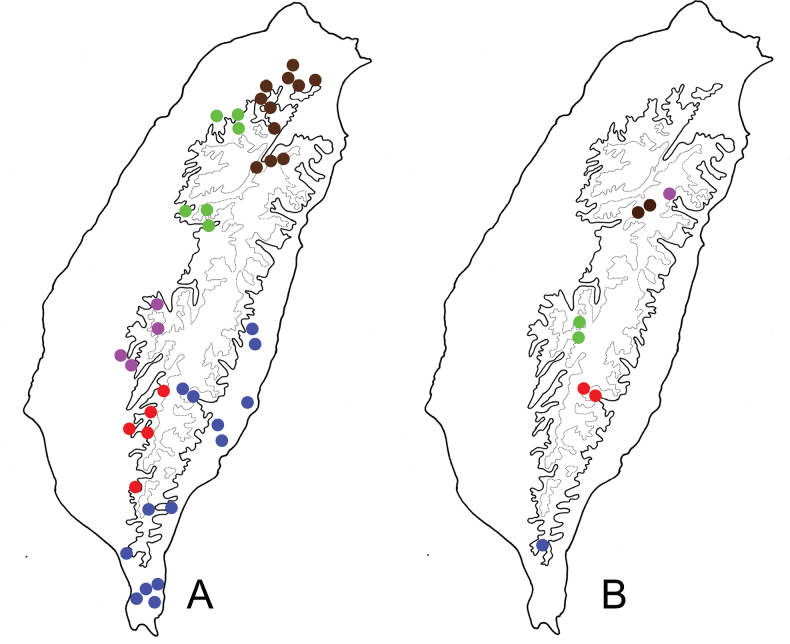
Distribution of *Paraplotes* species of Taiwan, solid line: 1000 m, broken line: 2000 m **A** widespread species, *P. yuae* indicated by green spots, *P. taiwana* indicated by blue spots **B** localized species, different colors represent different species (details in Lee 2015)

Species of *Paraplotes* are multivoltine. Adults are nocturnal and closely associated with their Urticaceae host plants (*Pilea* spp. (Fig. 4C) and *Lecanthus peduncularis* (Wall. ex Royle) Wedd.). These plants are widely distributed and grow on the edges of forests and along roadsides (Fig. 4A), walking trails, and rivers. These environments are easily accessible, hence adults can be collected by searching host plants at night. Females deposit eggs on the roots of plants (Fig. 4E). Larvae feed on young leaves and soft shoots (Fig. 4F). Mature larvae leave the host plant and burrow into the soil, where they build chambers underground for pupation. Duration of immature stages is typically less than one month. Collections were made by searching for adults on host plants at night. More than 500 specimens were collected from all over Taiwan (Lee 2015). Adults of *Paraplotes* were also collected using Malaise traps installed around host plants. A few adults of *P. taiwana* were collected using Malaise traps in Tulanshan (Taitung County). Adults were collected during all months except January, February, and March, with peak abundance during late June to late August. This species is limited to lowlands no higher than 1500 m (Fig. 5A).

**Figure 6.**
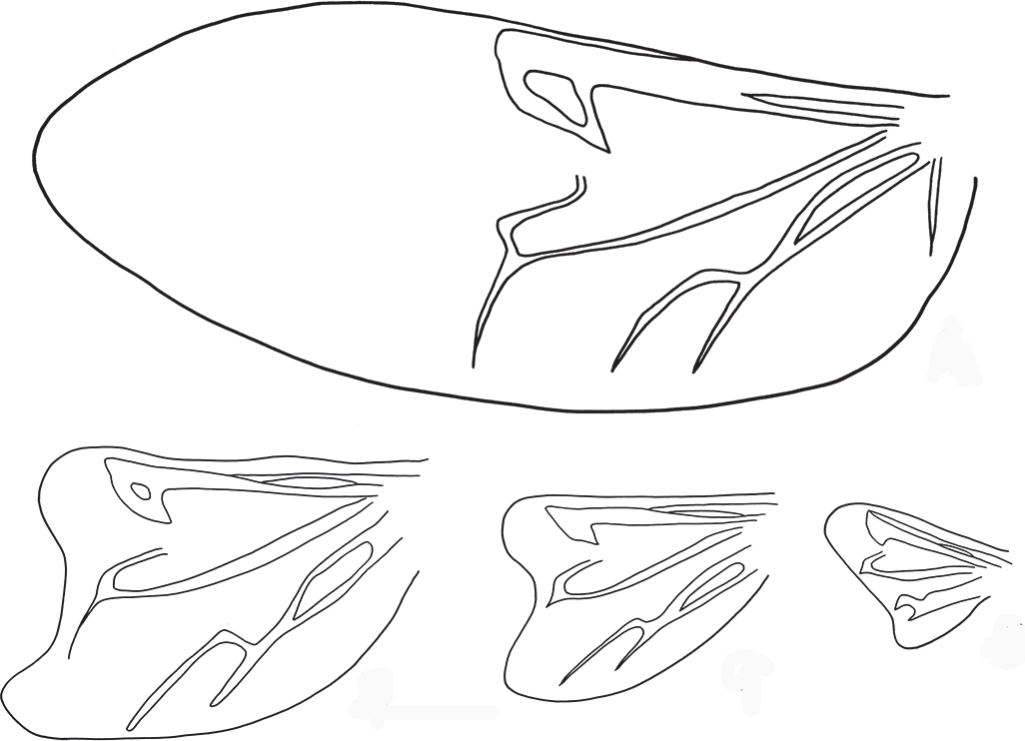
*Paraplotes yuae*, hindwing **A** male **B** female, Wushihkeng (烏石坑) **C** female, Anmashan (鞍馬山) **D** female, Wuchihshan (五指山); all hindwing in same scale (Lee 2015)

Ten species of *Paraplotes* are recognized in Taiwan. Five of these are widespread and inhabit lowlands (Fig. 5A), but the other half are restricted to a few localities in mountainous areas (Fig. 5B). Reduction of hind wings in females varies greatly among different populations, although most females in the same populations have similar hind wing lengths. For example, the range of variation of reduction of hindwings is extreme in populations of *P. yuae* (Fig. 6B–D) in northwest Taiwan (Fig. 5A). Females collected from Wushihkeng (烏石坑) have the least reduced hind wings (38%) (Fig. 6B). In contrast, reduction is more extreme in Wuchihshan (五指山) (18%) (Fig. 6D). Such variations may result from the occasional occurrence of diurnal behavior in the population at Wushihkeng. More than 10 males of *P. yuae* were observed gathering and feeding on leaves of Dumasia villosa subsp. bicolor (Hayata) Ohashi & Tateishi (Fabaceae) (Fig. 4D) during the day on July 13, 2008. However, an additional 15 adults were collected at night on another occasion (March 21, 2013). Thus, the reduction of hind wings seems to be promoted by the change from diurnal to nocturnal habits. Based on Southwood’s assumption (1962), diurnal habits are environmentally unstable (risky) compared to nocturnal habits.

### ﻿Genus *Shairella* Chûjô – a case of reduction of hindwings resulting from adaptations to mountainous habitats

**Figure 7.**
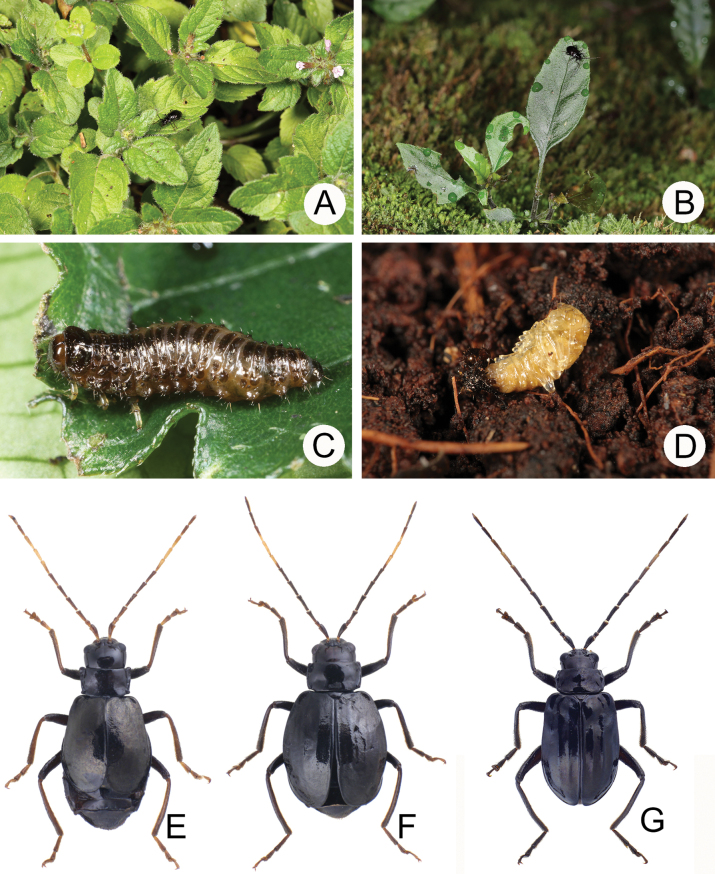
The genus *Shairella* Chûjô **A**Clinopodium laxiflorum var. taiwanianum (Lee & Beenen, 2017) **B***Hemiboea bicornuta* (Lee, 2022) **C** Larva of *S. guoi* feeding on leaves of *Strobilanthes flexicaulis***D** pupa of *S. guoi***E** Adult of *S. aeneipennis* species group (Lee & Beenen, 2017) associated with C. laxiflorum var. taiwanianum**F** adult of *S. cheni* species group (Lee and Beenen 1027) associated with *H. bicornuta***G** adult of *S. quadricostata* (Lee 2022) associated with *H. bicornuta*.

**Figure 8.**
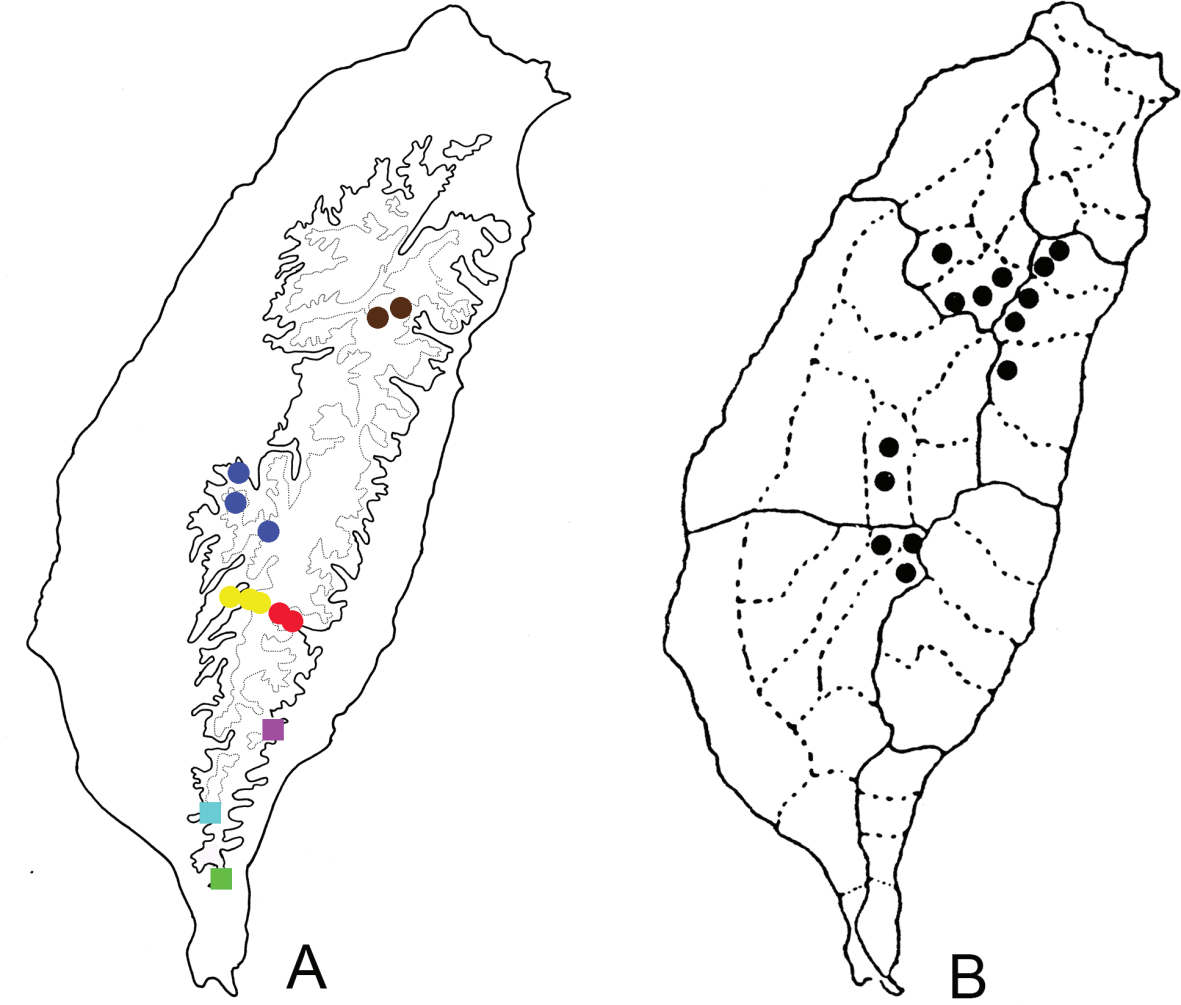
Distribution of brachelytrous *Shairella* species and Clinopodium laxiflorum var. taiwanianum**A** solid line: 1000 m, broken line: 2000 m; different colors represent different species, circles represent species associated with C. laxiflorum var. taiwanianum, squares represent species associated with *H. bicornuta***B**C. laxiflorum var. taiwanianum (after Lee and Beenen 2017).

This genus was considered brachelytrous and monotypic when Chûjô (1962) erected it. Adults are nocturnal and closely associated with their host plants: Clinopodium laxiflorum var. taiwanianum Hsieh & Huang (Fig. 7A) (Lamiaceae), *Strobilanthes flexicaulis* Hayata (Acanthaceae), and *Hemiboea bicornuta* (Hayata) Ohwi (Fig. 7B) (Gesneriaceae). These plants are widely distributed and grow on the edges of forests and along roadsides, walking trails, and rivers. These environments are easily accessible; hence, adults could be collected by searching host plants at night. *Shairella* species appear to be univoltine based on field observations (unpublished data TCRT). Larvae are diurnal and found on the host plant’s leaves during late February. They are easily discovered when feeding on the tops of leaves during the day (Fig. 7C). Larval development takes about one month, based on laboratory rearing. Mature larvae leave the host plant and burrow into the soil, where they build underground chambers for pupation. Pupal stage (Fig. 7D) duration is about 20 days, and adults begin to emerge after early April. Adults are nocturnal and have been observed in the field from May to August. Approximately 240 specimens have been collected throughout Taiwan (Lee and Beenen 2017). The distributions of *Shairella* species seem to be determined by their host plant. They can be separated into two species groups based on morphology and host plants. Adults of the *S. aeneipennis* group with noticeably reduced elytra (Fig. 7E) are associated with Clinopodium laxiflorum var. taiwanianum Hsieh & Huang (Lamiaceae) (Fig. 8A). This plant grows in middle elevations (1500– 2500 m) in central Taiwan (Fig. 8B). Four members of this group (Fig. 8A) are monophagous and their distributions coincide with the distribution of the host plant (Fig. 7B). The *S. cheni* group with less reduced elytra (Fig. 7F) includes three species in southern and southeastern Taiwan (Fig. 8A), which is out of the range of Clinopodium laxiflorum var. taiwanianum (Fig. 8B). The host plant of these species is *Strobilanthes flexicaulis* Hayata (Acanthaceae) and *Hemiboea bicornuta* (Hayata) Ohwi (Gesneriaceae) (Fig. 7B), which are abundant at low elevations.

**Figure 9.**
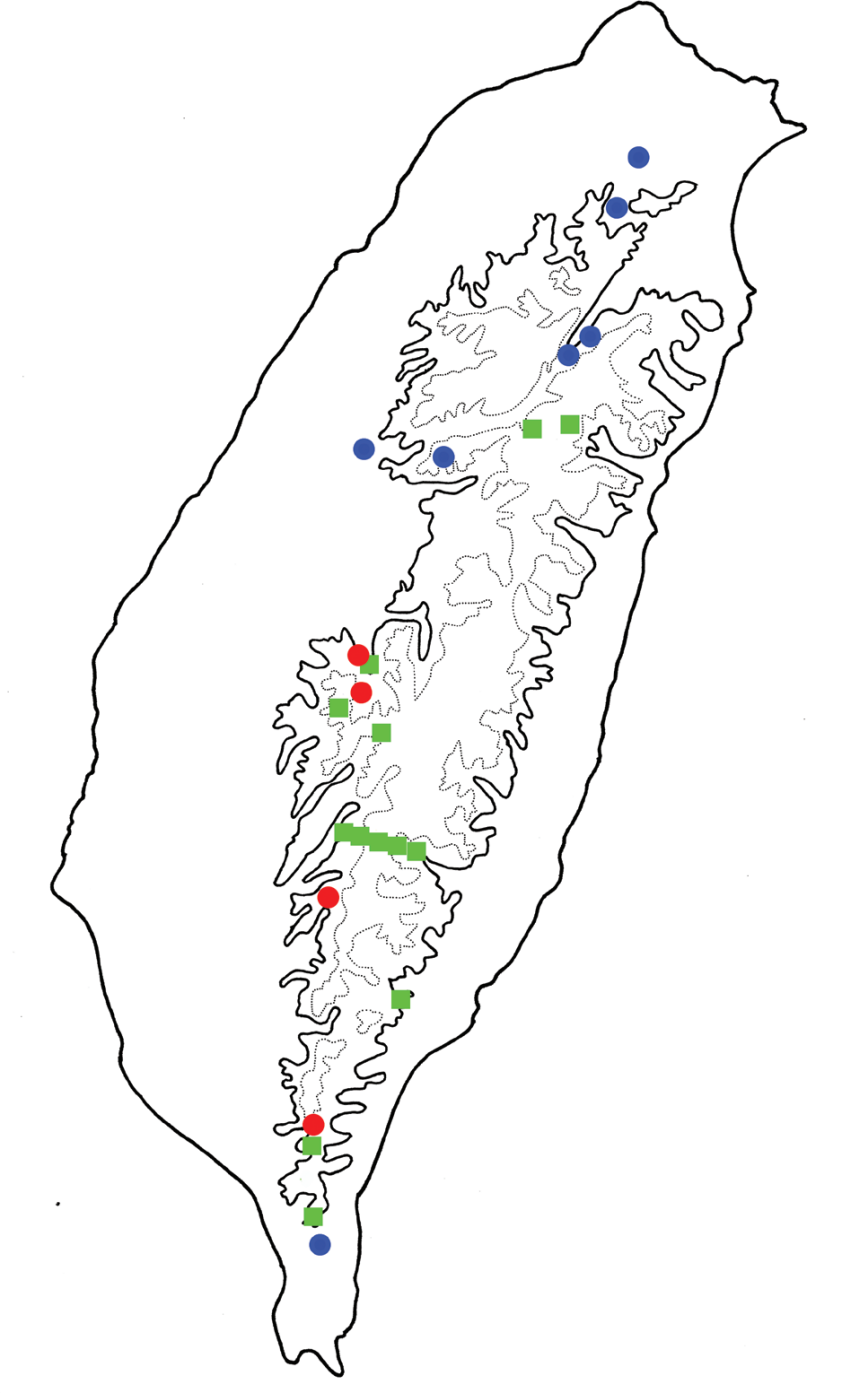
Distribution of *Shairella quadricostata* and brachelytrous *Shairella* species, solid line: 1000 m, broken line: 2000 m. Green squares, brachelytrous species; blue circles, adults of *S. quadricostata* with normal hindwings; red circles adults of *S. quadricostata* with reduced hindwings.

**Figure 10.**
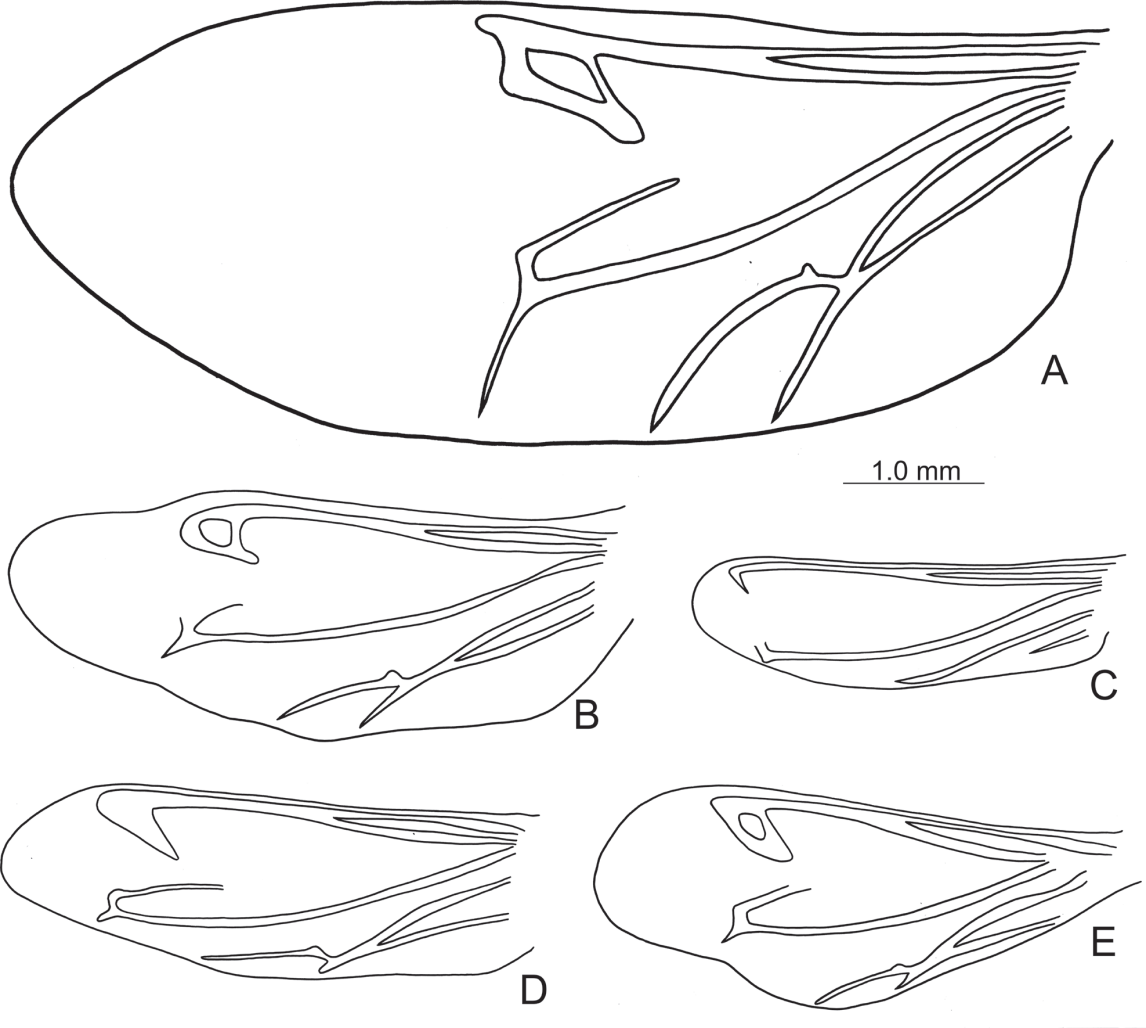
Hindwings of *Shairella quadricostata***A** female, from Wulai (烏來) **B** female, from Tengchih (藤枝) **C** female, from Erhwanping (二萬坪) **D** female, from Hsito (溪頭) **E** male, from Peitawushan (北大武山). (Lee 2022)

*Shairella quadricostata* (Kimoto, 1996) (Fig. 7G) was transferred from *Japonitata* with normal elytra, and adults feed on leaves of *H. bicornuta* (Lee 2022). Hindwings are normally developed (Fig. 10A) in northern and central Taiwan and low-elevations of southern Taiwan (Fig. 9), but they are reduced to different degrees between different populations of mid-elevations of southern Taiwan (Figs 9, 10B–E). They are allopatric relative to other members of the genus except at Erhwanping (二萬坪) and Hsitou (溪頭), where *S. aeneipennis* also occurs (Fig. 9). However, they are separated ecologically since both species utilize different food plants (*H. bicornuta* for *S. quadricostata* and C. laxiflorum var. taiwanianum for *S. aeneipennis*). Adults of *S. hsiehae* Lee & Huang, 2023 were collected from Peitawushan (北大武山) on the same trail as *S. quadricostata*. However, they occurred at higher altitudes (above 1600 m), while adults of *S. quadricostata* were found at localities below 1000 m. Thus, both species are presumably allopatric, occurring at different altitudes.

Adults of *S. quadricostata* (Fig. 7G) possess long antennae and a darker color and are adapted to nocturnal activity. Some populations have reduced hindwings (Fig. 10), presumably as an adaptation to stable microhabitats (mid-altitudes in south Taiwan) based on Southwood’s assumption (1962) (Fig. 9). Mountains represent historically persistent habitats, because populations can move upward or downward to minimize the impact of climatic fluctuations. Mountains are also isolated and represent an energetically costly environment for flight (Wagner and Liebherr 1992). In some *Shairella* species, elytra are reduced (Fig. 7F) due to allopatric speciation in southern Taiwan (Fig. 8A). Possibly, the shift in host plants resulted in adaptive radiation, leading to reduced elytra (Fig. 7E) in mid-altitudes of central Taiwan (Fig. 8A).

### ﻿Genus *Taiwanoshaira* Lee & Beenen – a case of reduction of hindwings resulting from adaptation to moss cushion habitats

Moss (Bryophyta) cushions constitute a special environment characterized by several important features: food source and habitat for overwintering when many tracheophytes are absent; buffered temperature and moisture; and the small size of spaces among the stems and leaves within cushions (Glime 2006). Based primarily on taxonomic surveys, various habitats have been cited as having inordinately high frequencies of flightless insects (Roff 1990), but this kind of habit is omitted. Only 52 leaf beetle species from 15 genera are documented to live within moss cushions (bryobionts). All moss-inhabiting leaf beetles belong to the tribe Alticini, a group of about 12,000 species worldwide (Konstantinov et al. 2013). Of this group, some species of *Benedictus* Scherer, *Cangshanaltica* Konstantinov et al., *Ivalia* Jacoby, *Paraminota* Scherer, *Paraminotella* Döberl and Konstantinov, and *Phaelota* Jacoby are known to occur inside moss cushions or leaf litter in various regions of Asia. These species are characterized by small, round bodies and the absence of wings (Döberl and Konstantinov 2003; Konstantinov et al. 2013; Damaška and Konstantinov 2016; Damaška and Aston 2019; Damaška et al. 2020, 2022; Ruan et al. 2020, 2022, 2023; Takemoto and Suenaga 2021).

**Figure 11.**
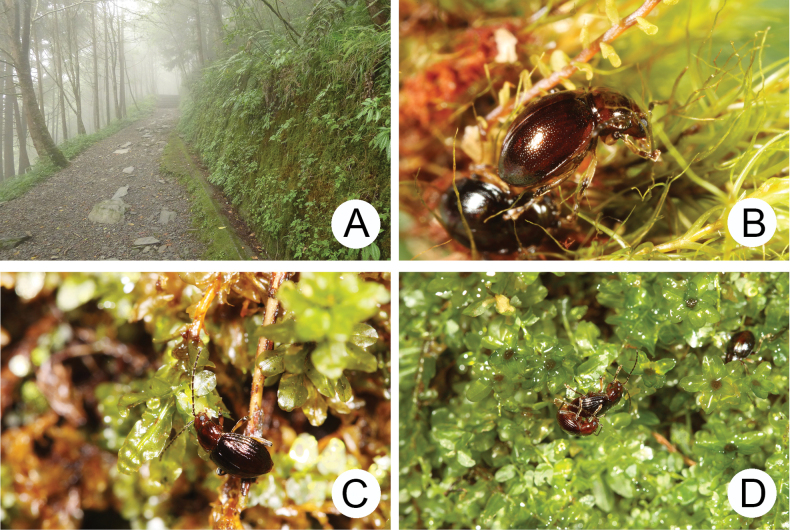
The genus *Taiwanoshaira* Lee & Beenen **A** microhabitat for *T. taipingshanensis* and *T. tsoui* Lee & Beenen at Yuanyang Lake (鴛鴦湖) **B** active adults of *T. chujoi* inside moss cushions at Pilu (畢祿) **C** adult of *T. taipingshanensis* feeding on leaves of *Plagiomnium vesicatum* at Yuanyang Lake (鴛鴦湖) **D** adults of *T. taipingshanensis* mating at Yuanyang Lake (鴛鴦湖) (after Lee and Beenen 2020).

**Figure 12.**
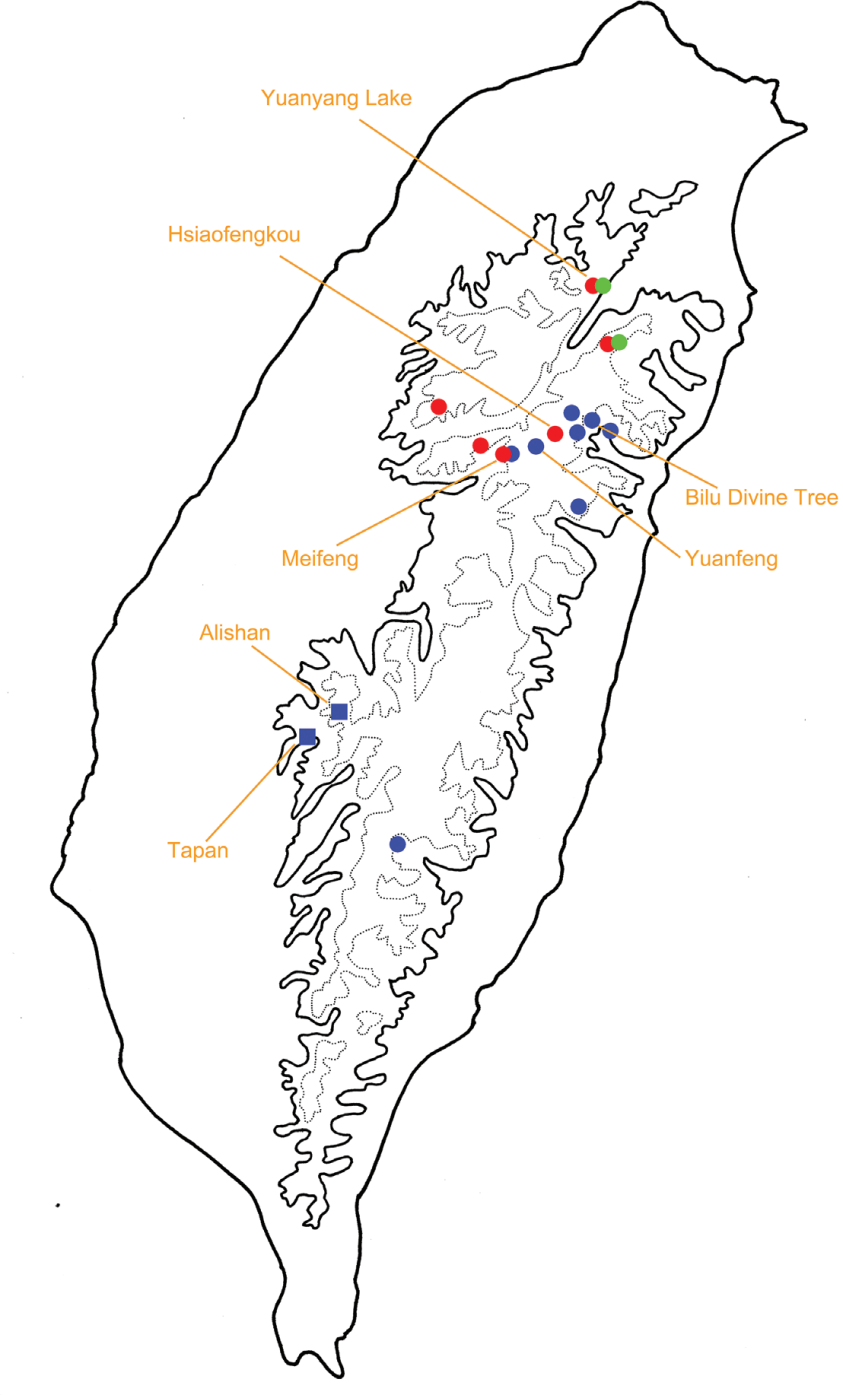
Distribution of *Taiwanoshaira* species, solid line: 1000 m, broken line: 2000 m. Blue dots, *T. chujoi*; blue squares, historical records for *T. chujoi*; red dots, *T. tsoui*; green dots, *T. taipingshanensis* (after Lee and Beenen 2020).

The genus *Taiwanoshaira* is the first genus of moss-feeding Galerucinae s. str. described that is endemic to Taiwan (Lee and Beenen 2020). Adults are nocturnal, with feeding and mating in moss cushions at night (Fig. 11). Adults were collected in Malaise traps set at specific localities such as Meifeng (梅峰), Yuanfeng (鳶峰), Hsiaofengkou (小風口), and Bilu Divine Tree (碧綠神木) (Fig. 12). During visits to these localities, more than 150 specimens were captured by hand-collecting at night.

Mosses are common all over Taiwan due to high humidity and precipitation. They are most dominant in cloud forests, which cover most montane areas above 1000 m altitude. However, species of *Taiwanoshaira* are restricted to limited areas, based on the TCRT’s collecting experience (Fig. 12). Moreover, they were absent at some localities where they were recorded 40 years ago, such as Tapan (達邦) and Alishan (阿里山) for *T. chujoi*. They are currently common in only a few places, including Yuanyang Lake (鴛鴦湖), Hsiaofengkou (小風口), and Bilu Divine Tree (碧綠神木). Of these localities, the climatic patterns of the cloud forest at Yuanyang Lake (鴛鴦湖) were studied from 1994 to 2004 (Lai et al. 2006). This site (24°35'N, 121°24'E) is situated in Chi-Lan Mountain at an elevation of 1650 to 2420 m above sea level, where the annual mean air temperature is 12.7 °C and the relative humidity exceeds 90%. The annual precipitation varied between 2109 mm (in 1995) and 4727 mm (in 2001), with an average of 3396 mm, spread over 239 mean rainy days per year. These occurrences indicate that *Taiwanoshaira* species favour microhabitats with high humidity year-round. Large moss cushions provide moist microclimates without large fluctuations and thus represent a stable and largely predictable habitat.

### ﻿Genus *Sikkimia* Duvivier – a case for the effect of sexually dimorphic antennae on survival and speciation of wingless species

**Figure 13.**
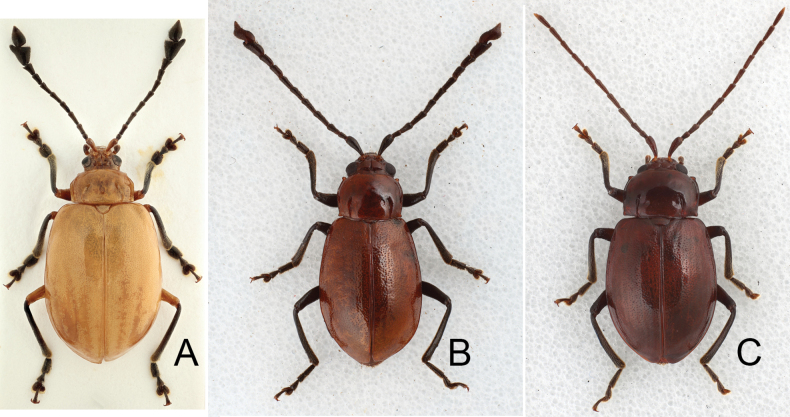
Males of *Sikkimia* species, dorsal view **A***S. rufa* (China) **B***S. sufangae* (Taiwan) **C***S. tsoui* (Taiwan) (Lee and Bezděk 2016).

**Figure 14.**
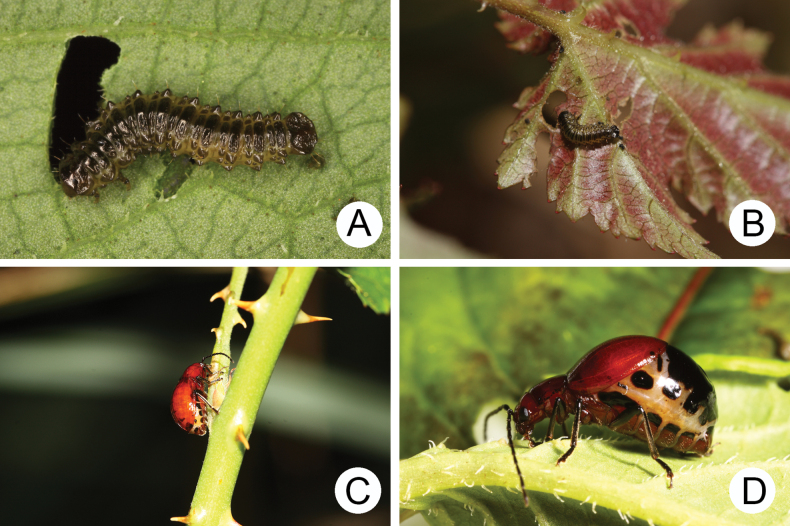
The genus *Sikkimia* Duvivier **A** larva of *S. sufangae* feeding on *Polygonum chinense***B** larva of *S. tsoui* feeding on *Rubus corchorifolius***C** female of *S. tsoui*. feeding on leaves of *R. swinhoei***D** female of *S. sufangae* (Lee and Bezděk 2016).

Although *Sikkimia* is widespread in continental Asia and Taiwan, only Taiwanese species are wingless and nocturnal (Lee and Bezděk 2016) (Fig. 13). Taiwanese species of *Sikkimia* appear to be univoltine, based on field observations. Larvae are nocturnal and found on the underside of leaves between February and April. Larval development took 20–22 days under laboratory conditions (Fig. 14A, B). Mature larvae left the host plant and burrowed into the soil, where they built underground chambers for pupation. The pupal stage lasts about 22 days, and adults begin to emerge after April. Adults were nocturnal and lived for more than three months, a lengthy life span for chrysomelids. Presumably, *Sikkimia* species overwintered as adults, as some females were collected during winter. Adults and larvae were closely associated with the host plant *Polygonum chinense* L. (Fig. 14A). This plant is widely distributed and grows on the edges of forests, roadsides, walking trails, and rivers. The northern species, *Sikkimia tsoui* Lee & Bezděk, feeds on *Rubus* species (Rosaceae). Since these habitats are easily accessible, adults can be collected by searching on host plants at night. Approximately 350 specimens were collected all over Taiwan.

Adults of Taiwanese species are characterized by a reduction of the hindwings and elytral humeral calli (Fig. 13B, C). The reduction of hind wings may result from the production of physogastric females (Fig. 14D). Nocturnal behavior presumably increases survival since natural enemies are less of a threat. As observed in adverse environments such as islands, deserts and alpine regions, flight is not essential at night, and energy can be delivered to egg production, as suggested by Beenen and Jolivet (2008).

**Figure 15.**
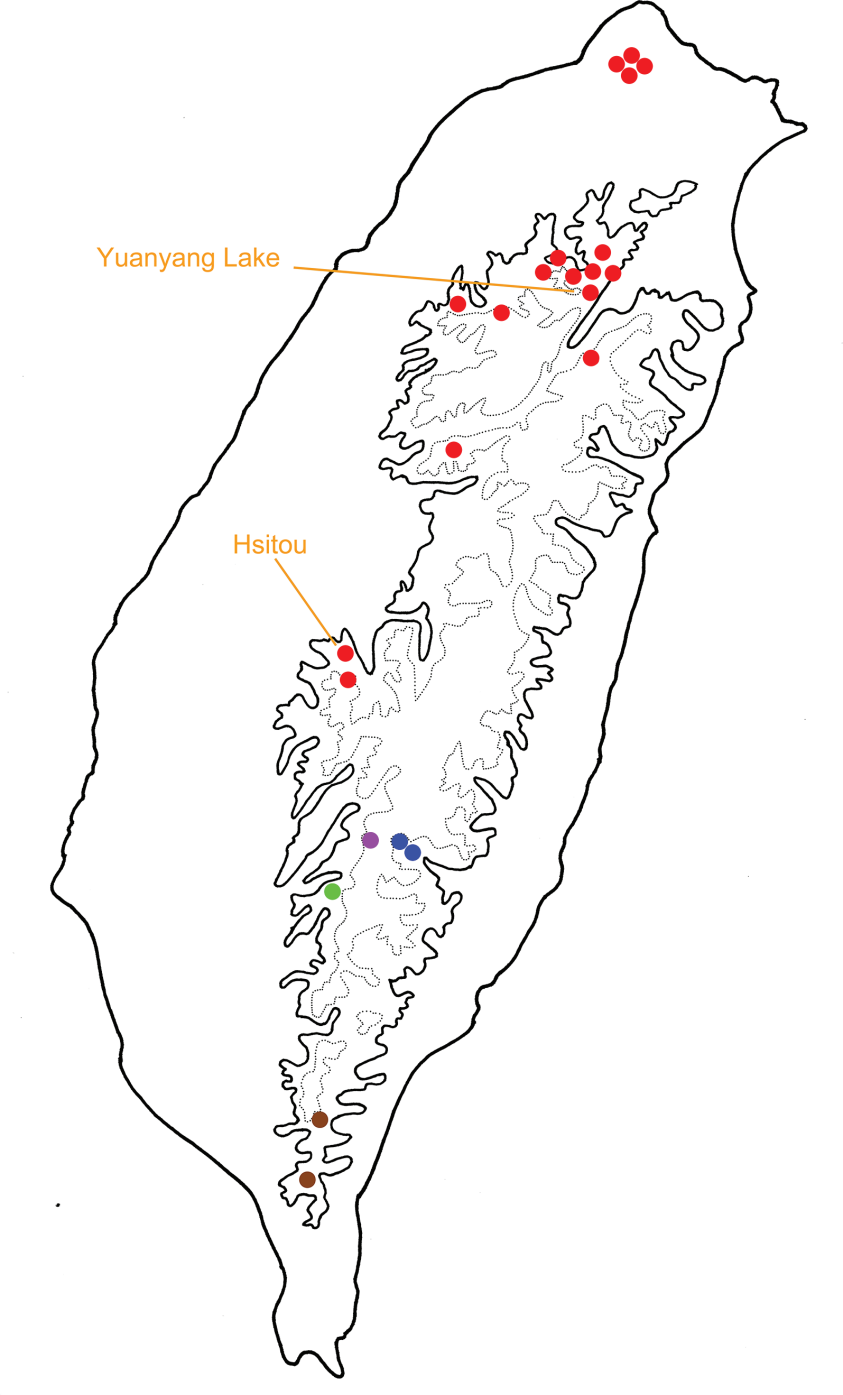
Distribution of *Sikkimia* species of Taiwan, solid line: 1000 m, broken line: 2000 m; different colors represent different species. Red dots, *S. tsoui* (after Lee and Bezděk 2016)

All *Sikkimia* species are allopatric, restricted to different mountain ranges, and not separated by elevation. Populations of *S. tsoui* have a wide distribution and occupy northern and central Taiwan (Fig. 15). In addition to its wider distribution, populations of *S. tsoui* are abundant in some areas (increased fitness). For example, there were many adults at Hsitou (溪頭) and Yuanyang Lake (鴛鴦湖) with more than 50 individuals collected in one night. Both features may reflect the development of some unique characters in *S. tsoui*. Males of *S. tsoui* lack enlarged apical antennomeres (Fig. 13C), a character that may be involved in complex courtship behavior and the lack of this secondary sexual character may result in lower speciation across the wide range of distribution. Moreover, the ability to feed on a wide range of host plants (e.g., populations from Yangmingshan National Park (陽明山國家公園) were observed feeding on *R. swinhoei* Hance and *R. corchorifolius* L. f. (Rosaceae) (Fig. 14B, C). In addition, females with smaller sclerotized areas of abdominal tergites (Fig. 14C) represent plesiomorphic winged galerucines; those with larger, entirely sclerotized areas of abdominal tergites that provide better protection (Fig. 14D) represent a derived condition.

### ﻿Genus *Furusawaia* Chûjô – a case of aposematic spots on elytra with bizarre behavior

**Figure 16.**
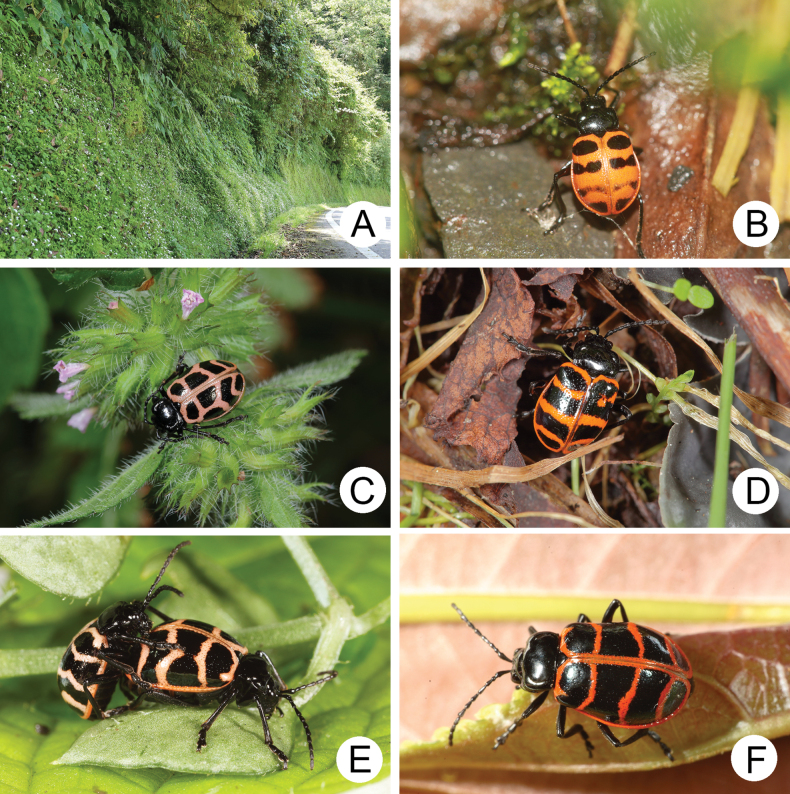
The genus *Furusawaia* Chûjô **A** microhabitat of *F. lui* in Hsinpaiyang (新白楊) **B** adult of *F. jungchani* in the daytime, Huakang (華崗) **C** adult of *F. lui* at night, Hsinpaiyang (新白楊) **D** adult of *F. tahsiangi* in the daytime, Hsuehshan (雪山) **E** two adults of *F. tsoui* Lee & Bezdèk at night, Jianqing trail (見晴步道) **F** adult of *F. yosonis* at night, Alishan (阿里山) (Lee and Bezděk 2021).

*Furusawaia* Chûjô is a small wingless galerucine genus distributed in China (two species) and Taiwan (five species). Most members of wingless genera are exclusively nocturnal. However, adults of *Furusawaia* exhibit bizarre behavior with diurnal or nocturnal habits in different individuals of the same species. Adults can often be observed walking on forest trails during the day, while others are active at night. Such behavior may be associated with bicolored elytra (Fig. 16), which may be shared with those bicolored, wingless galerucines, but further study for confirmation is required. Although their food plants are known (*Stellaria* species (Caryophyllaceae); Fig. 16A), collecting adults by searching food plants at night is difficult. Only 96 specimens were available for the study (Lee and Bezděk 2021).

**Figure 17.**
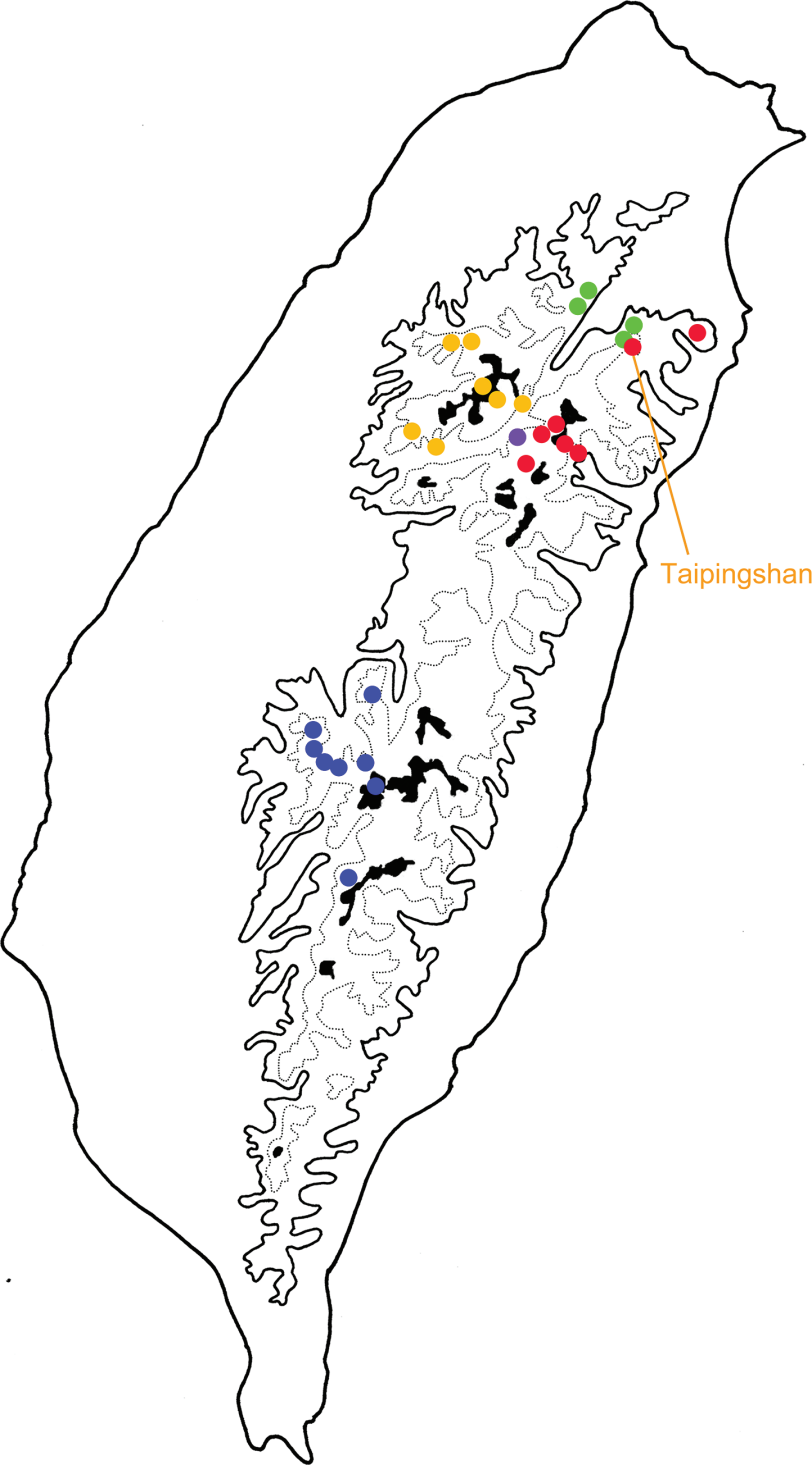
Distribution map of *Furusawaia* species in Taiwan, solid line: 1000 m, broken line: 2000 m, black areas: 3000 m; different colors represent different species (after Lee and Bezděk 2021).

In Taiwan, adults were observed walking or resting on forest trails at low altitudes (1000–2000 m) in northern Taiwan or middle and high altitudes (above 2000 m) in central and southern Taiwan. Most *Furusawaia* species have broad distributions except for *F. jungchani* Lee & Bezděk. Two species are sympatric in Taipingshan (太平山). Most adults were collected at lower altitudes (below 2500 m), where they are easily accessible to collectors. Very few individuals were found above 3000 m (Fig. 17).

### ﻿Genus *Apterogaleruca* Chûjô and *Hirtigaleruca* Chûjô – a case of well-adapted brachelytrous galerucines in Taiwan

**Figure 18.**
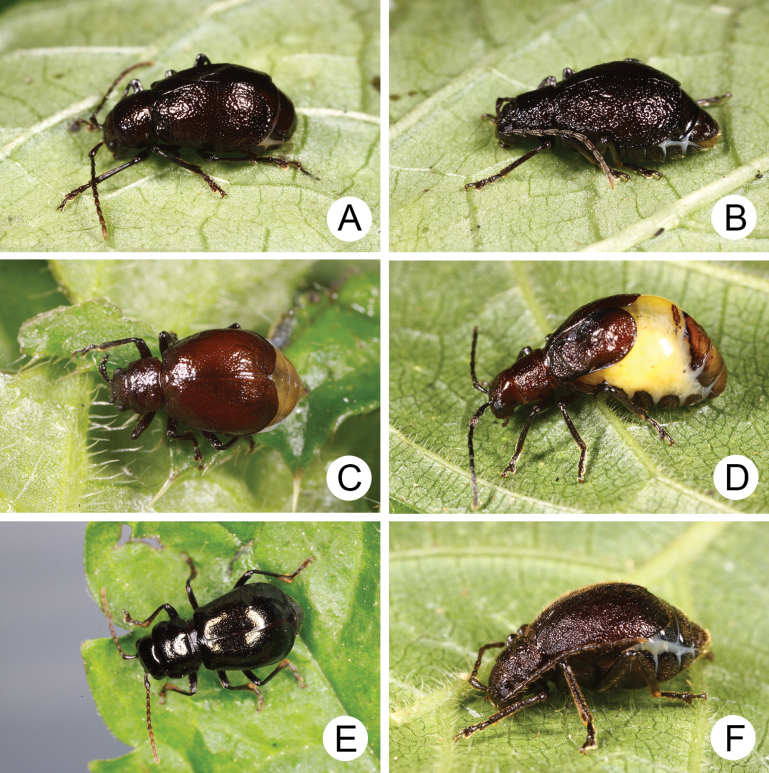
Adults of *Apterogaleruca* and *Hirtigaleruca***A***A. hirtihumeralis*, male, collected from Tengchih (藤枝) **B** different individual collected at same locality **C** same species, feeds on Cirsium japonicum var. australe, collected from Yangmingshan (陽明山) **D** same species, female, collected from Neiwan (內灣) **E***A. uenoi*, male, collected from Pilu (畢祿) **F***Hirtigaleruca aptera*, male, collected from Ima (依麻).

**Figure 19.**
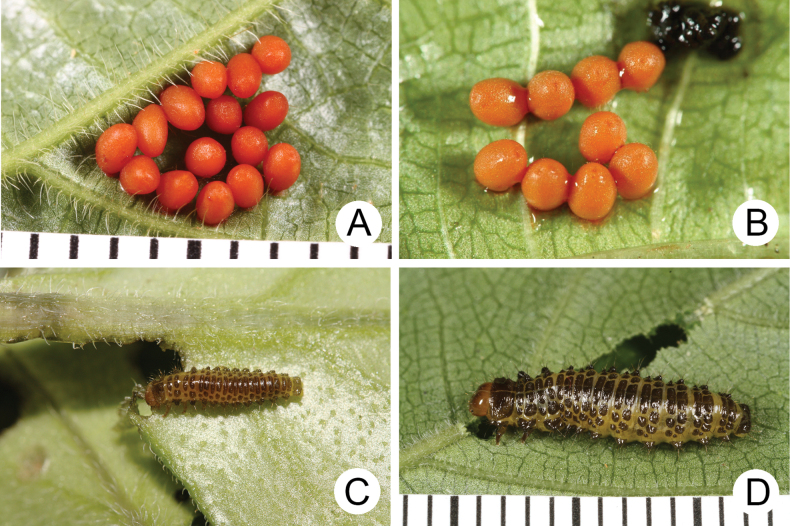
Immature stages of *Apterogaleruca* and *Hirtigaleruca***A** eggs of *A. hirtihumeralis***B** eggs of *Hirtigaleruca aptera***C** second-instar larva of *A. hirtihumeralis* feeding on Cirsium japonicum var. australe, collected from Nanya (南雅) **D** third-instar larva of *A. hirtihumeralis* feeding on Elatostema lineolatum var. majus, collected from Huisun (惠蓀).

*Apterogaleruca* comprises two species, *A. hirtihumeralis* Chûjô and *A. uenoi* Kimoto. The former represents a species group that is widespread from lowlands to 2000 m (Fig. 18A–D). Adults and larvae feed on leaves of various species of Urticaceae, including *Boehmeria densiflora* Hook. & Arn., Elatostema lineolatum Wight var. majus Wedd. (Fig. 18D), *E. platyphyllum* Wedd., *Gonostegia hirta* (Blume) Miq., *Pellionia radicans* (Siebold & Zucc.) Wedd., *Pe. scabra* Benth., *Pilea angulata* (Blume) Blume, *Pi. melastomoides* (Poir.) Wedd., *Pi. rotundinucula* Hayata, *Urtica thunbergiana* Siebold & Zucc., and some species of Asteraceae such as Cirsium japonicum DC. var. australe Kitam. (Figs 17C, 18C) and Gynura divaricata (L.) DC. subsp. formosana (Kitam.) F.G. Davies. Members of *Apterogaleruca* are multivoltine and occur at more localities compared to other wingless galerucines. Physogastric females lay 16–18 eggs in a single egg mass (Fig. 19A). The larvae hatch in three to five days. Larvae feed on leaves, and the larval duration is ~24−26 days. Mature larvae (Fig. 19D) crawl into the soil and build underground chambers for pupation. The pupal stage lasts five to eight days. *Apterogaleruca uenoi* represents a species group that occurs above 2000 m. Adults of the *A. uenoi* group (Fig. 18E) differ from those of the *A. hirtihumeralis* group by possessing smooth bodies and short antennae. *Hirtigaleruca* is a monotypic genus (type species: *H. aptera* Chûjô) that inhabits southern Taiwan. This genus may prove to be a species group within *Apterogaleruca* because of similar morphologies of adults, except for dense hairs on the elytra (Fig. 18F). Moreover, immature stages of *Hirtigaleruca* are similar to those of *Apterogaleruca* and share the same host plants with the exception of having fewer eggs in a single egg mass (Fig. 19B). Observations of two or three species of the same species group or different groups at the same locality is typical.

Unlike most wingless members of Galerucini in Taiwan, individuals of *Apterogaleruca* inhabit not only mountainous regions but also alpine regions and lowlands. Members of this genus are most widespread and utilize most ecological niches in Taiwan since they are multivoltine and polyphagous in lowlands, and modifications of their external morphology (smooth surface and short antennae) are adapted to alpine regions.

## ﻿Discussion

Cox (2004) described the principles of various traps and species diversity of trapped leaf beetles. Malaise traps perform poorly for beetles because many adults tend to drop when striking an obstacle. For flightless leaf beetles, only apterous/brachypterous flea beetles were collected using flight interception traps. Ruan et al. (2023) indicated that apterous flea beetles were also collected using ethanol pan traps. In this study, adults of *Lochmaea* Weise, *Paraplotes* Laboissière, and *Taiwanoshaira* Lee & Beenen were successfully collected using Malaise traps. This result implies that adults of these genera crawl up the traps during nocturnal activity.

Evolutionary scenarios for reduction of hindwings in each galerucine genus in Taiwan are different and characteristic. Beenen and Jolivet (2008) indicated that brachyelytry seems to occur in harsh environments but only when conditions are stable. This seems consistent for wingless species of *Lochmaea* inhabiting alpine zones and *Taiwanoshaira* inhabiting moss cushions. Southwood (1962) indicated that flightless species are associated with permanent habitats and flying species with temporary, environmentally changing, or unstable habitats. Thus, nocturnal habits are more environmentally stable compared to diurnal habit since nocturnal habits are represented by all wingless galerucines. Moreover, physogastric females are apt to lose their hind wings after adapting to nocturnal habits (*Paraplotes*). For nocturnal galerucines, mountainous habitats (1000–2500 m) are more stable and promote the reduction of the hind wings and elytra (*Shairella*). Dimorphic sexual characters may still exist after hind wings are reduced, but when those characters are lost, populations may increase while species richness decreases (*Sikkimia*). The nocturnal, wingless galerucines may regain diurnal habitats, with the corresponding appearance of aposematic markings on the body (*Furusawaia*). Wingless galerucines may become more widespread if they are multivoltine and polyphagous (*Paraplotes* and *Apterogaleruca*). Adaptation of wingless galerucines to alpine zones may be accompanied by modification of external morphology (smooth bodies and short antennae) (*Apterogaleruca*).

## ﻿Additional information

### Author ORCIDs

Chi-Feng Lee https://orcid.org/0000-0003-1996-0557

### Data availability

All of the data that support the findings of this study are available in the main text.
